# Total variation denoising-based method of identifying the states of single molecules in break junction data

**DOI:** 10.1186/s11671-024-03963-4

**Published:** 2024-01-29

**Authors:** Yuki Komoto, Jiho Ryu, Masateru Taniguchi

**Affiliations:** 1 SANKEN, Osaka University, 8-1 Mihogaoka, Ibaraki, Osaka 567-0047 Japan; 2 Artificial Intelligence Research Center, Osaka University, 8-1 Mihogaoka, Ibaraki, Osaka 567-0047 Japan; 3 Integrated Frontier Research for Medical Science Division, Institute for Open and Transdisciplinary Research Initiatives (OTRI), Osaka University, 8-1 Mihogaoka, Ibaraki, Osaka 567-0047 Japan

## Abstract

Break junction (BJ) measurements provide insights into the electrical properties of diverse molecules, enabling the direct assessment of single-molecule conductances. The BJ method displays potential for use in determining the dynamics of individual molecules, single-molecule chemical reactions, and biomolecules, such as deoxyribonucleic acid and ribonucleic acid. However, conductance data obtained via single-molecule measurements may be susceptible to fluctuations due to minute structural changes within the junctions. Consequently, clearly identifying the conduction states of these molecules is challenging. This study aims to develop a method of precisely identifying conduction state traces. We propose a novel single-molecule analysis approach that employs total variation denoising (TVD) in signal processing, focusing on the integration of information technology with measured single-molecule data. We successfully applied this method to simulated conductance traces, effectively denoise the data, and elucidate multiple conduction states. The proposed method facilitates the identification of well-defined plateau lengths and supervised machine learning with enhanced accuracies. The introduced TVD-based analytical method is effective in elucidating the states within the measured single-molecule data. This approach exhibits the potential to offer novel perspectives regarding the formation of molecular junctions, conformational changes, and cleavage.

**Supplementary Information:**

The online version contains supplementary material available at 10.1186/s11671-024-03963-4.

## Introduction

Single-molecule conductance measurements have garnered significant attention in diverse applications ranging from the realization of molecular devices to the development of novel analytical techniques and the exploration of nanoscale physical properties [1–3]. Among the prominent techniques used in measuring single-molecule conductance are the mechanically controllable and scanning tunnelling microscope break junction (BJ) techniques based on the BJ method [4–6]. This method involves breaking the atomic contacts of metals to yield metal nanogap electrodes. When a molecule forms a bridge between the nanogap electrodes, the resulting tunneling current manifests as a conductance plateau in the measurement trace. Accurately discerning the conductance state of the single molecule using this trace is crucial in detecting chemical reactions[7–9] and identifying deoxyribonucleic acid nucleobases within these nanogaps[1, 10, 11]. However, unlike conventional methods that analyze multiple molecules, the BJ method focuses on measuring the conductance of a single molecule. Consequently, this measurement is highly susceptible to external noise and fluctuations in the molecular junction structure, leading to considerable variations in the observed conductance and impeding the analyses of individual traces containing single-molecule data [12–15]. Moreover, certain single-molecule junctions exhibit multiple conduction states owing to differences in the junction structures or molecular oxidation states [16–18]. The dynamics of single-molecule junctions remain inadequately understood because of the unreliability of analyzing individual conductance traces. To mitigate noise, the construction of conductance histograms is a typical analytical method employed in BJ studies [3, 4]. However, the histograms often display broad peaks attributable to substantial variations in the conductance traces, rendering differentiation between distinct conduction states challenging.

Recently, remarkable advancements in analytical techniques have been reported, particularly with respect to machine learning [19]. The integration of machine learning methodologies has progressively expanded to single-molecule measurements [10, 20–27]. Innovative analytical techniques, such as supervised machine learning and unsupervised clustering methods, have enabled the classification of various types of conductance traces. However, current methods of single-molecule signal analysis have yet to provide clear insights into individual conductance traces without relying on statistical signals. The application of novel signal processing and optimization techniques to single-molecule experimental data exhibits potential in detecting changes in the conduction state within a conductance trace. The aim of this study was to develop an analytical method of discriminating between different states based on established signal processing techniques. Our objective was to eliminate the reliance on statistical signals and derive precise data directly from individual conductance traces.

## Method

### Total variation denoising

**Fig. 1:**
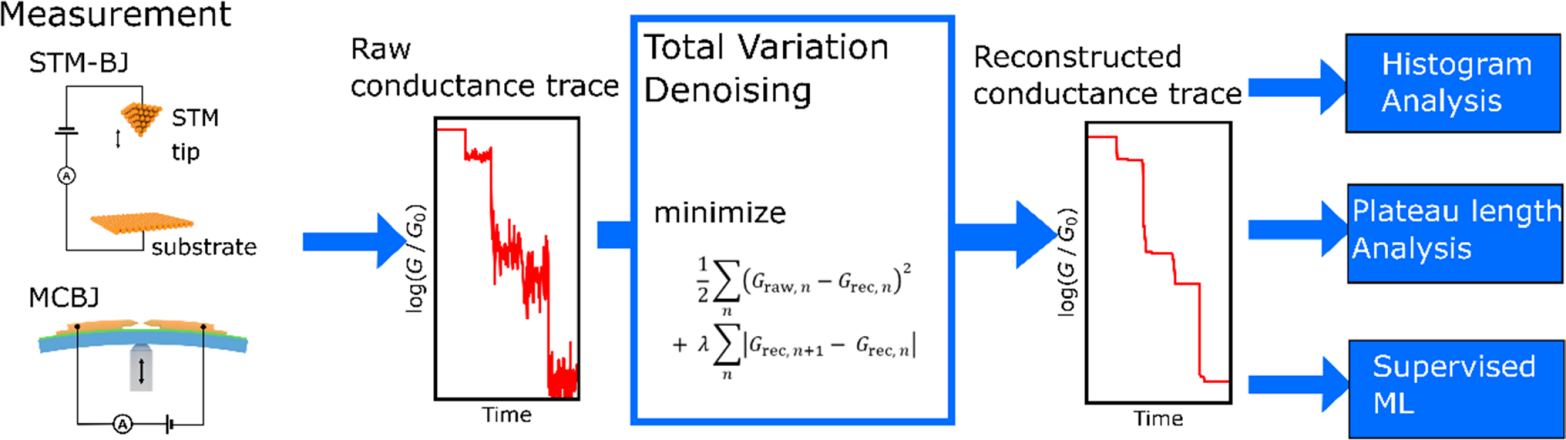
Schematic flow of the proposed method. In this study, the simulated conductance traces for validation of this method were analyzed for validation

We propose total variation denoising (TVD)-based signal reconstruction as a novel method of analyzing single-molecule conductance traces. A schematic of the proposed method is shown in Fig. 1, and Eq. 1 is minimized in TVD[28, 29].

1 $$\frac{1}{2}\mathop \sum \limits_{n} \left( {G_{{{\text{raw}}, n}} - G_{{{\text{rec}}, n}} } \right)^{2} + \lambda \mathop \sum \limits_{n} \left| {G_{{{\text{rec}}, n + 1}} - G_{{{\text{rec}}, n}} } \right|$$

G_raw, *n*_ and G_rec, *n*_ respectively represent the conductances at the *n*-th point in the raw and reconstructed conductance traces. In this study, differences in conductance were assessed using a logarithmic scale. For implementation, we employed the alternating direction multiplier method (ADMM) to minimize the nondifferentiable total variation[30, 31]. The analysis was performed with Python 3.10.9 with numpy package version 1.23.5, and scikit-learn version 1.2.1[32].

### Generation method of simulated conductance traces

In this study, the proposed analytical method was applied to simulated single-molecule traces. Three classes of simulated traces were generated as follows. First, the ground truth traces were generated. Plateaus conductance (log(*G*/*G*_0_), *G*_0_ is conductance quantum, 2*e*^2^/*h*, *e* and *h* are the elementary charge and Planck constant, respectively) and length were determined by normal distribution of center *G*, standard deviation σ*G*, and normal distribution of center *L*, standard deviation σ*L* in molecular conductance region. The parameters are shown in Table 1. Here, we assumed the conductance traces of typical small organic molecules in break junction measurement such as 4,4’-bipyridine, 1,4-benzenedithiol [4, 5, 12, 17, 33, 34]. These molecules have multiple conductance states [12, 17, 33, 34]. Some traces exhibit both conductance state and the other traces have only one state. For instance, 4,4’-Bipyridine has high- and low-conductance states with the log conductance difference of about 0.5 [17, 34]. We set σ*G* = 0.1 because the observed plateau conductance has a width of about 0.1 on the fluctuation log scale [13, 17, 34]. In single-molecule measurements, a two- or three-fold conductance plateau is often interpreted as a two- or three-molecule junction [4, 13, 18]. Therefore, it is reasonable to set the identical state with a log conductance difference smaller than log(2) = 0.3. For the plateau length, a plateau length *L* of 0.5–1 nm and a standard deviation σ*L* of 0.1 nm, which are typical lengths for small molecule BJ measurements, were set based on experimental results [4, 33]. The stretch length is set to 0.01 nm for data point. ﻿In metal contact region (*G* > *G*_0_), the plateaus consists of a plateau determined by a normal distribution with a probability of occurrence of 0.3, center 0.3, and standard deviation 0.02, and a plateau determined by a normal distribution with a probability of occurrence of 0.7, center 0, and standard deviation 0.01, the plateau length follows a normally distributed normal distribution with a center of 50 points and a standard deviation of 10 points for all class. Then, Gaussian noise was introduced the ground truth traces. The standard deviation is 0.1 in the metal junction region (*G* > *G*_0_) and 0.5 in other regions. 1000 traces were generated for three classes﻿.

**Table 1:** Parameter for generation of simulated conductance traces

	*G*	σ*G*	*L*/points	σ*L*/points
Class 1	− 2.5	0.1	50	10
	− 3	0.1	50	10
Class 2	− 2.5	0.1	100	20
Class 3	− 3	0.1	100	20

## Results and discussion

**Fig. 2:**
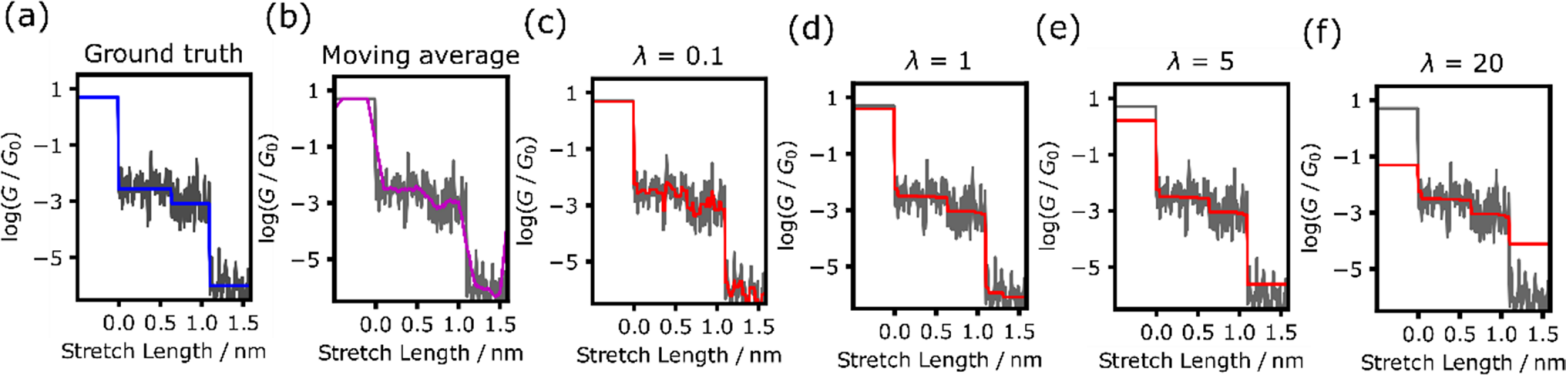
Reconstruction results of a simulated conductance trace. **a** Original trace. Blue line is noise-free ground truth. Gray line is generated by adding Gaussian noise to blue line. **b** Magenta line is reconstruction trace by moving average smoothing. **c**–**f** Red lines are reconstructed results by TVD analysis with λ = 0.1, 1, 5, and 20, respectively. All gray line is analyzed raw conductance trace

Equation 1 comprises two terms. The first term represents the *L*^2^ norm, indicating the Euclidean distance between the reconstructed and raw signals, signifying their proximity. The second term, which encompasses the sum of the differences between adjacent observation points, is referred to as the total variation. This term diminishes as the signal transitions towards smaller, flatter variations. The parameter λ, serving as the regularization parameter, influences the total variation, and higher λ values amplify the total variation, thus reducing the variation within the reconstructed trace. The results reconstructed using TVD for conductance traces at different λ values are shown in Fig. 2. The simulated conductance of Class 1 shown in Fig. 2a. Two conduction plateaus appears at log(*G*/*G*_0_) = –2.5 and –3. The presence of noise obscures the distinction between the two states in the signal. For comparison, Fig. 2b shows the trace reconstructed using simple moving average smoothing, which blurs the boundaries delineating the changes in conduction state while averaging to mitigate conductance variations. Moreover, accurate conductance determination is impeded by the conductances of the metal contacts. Figures 2c–f show the denoising results obtained via our proposed TVD-based method at various λ values. At smaller λ values, the dominance of the first term in Eq. 1 retains the similarity to the original signal (Fig. 2c). Conversely, larger λ values generally result in the overestimation of the step effect, aligning the conductances in the metal junction and post-molecular junction break regions more closely with the molecular conductance (Fig. 2f). However, higher λ values generally lead to the equalization of the changes in the molecular state. Notably, a clear depiction of two distinct steps emerges in the reconstructed trace at λ = 1. In this study, reconstructed traces with different λ in range 0.5–3 exhibit no difference in molecular conductance region as shown in Additional file 1: Fig. S1. Optimization of λ does not eliminate the difference from ground truth because the traces with noise were analyzed. In the analysis or real single-molecule data, there are no ground truth traces. In order to determine λ, the assumptions regarding the equivalence of distinct states of conductance is necessary. This assumption depends on the objectives underpinning the analysis. The plot showing the total variation loss as a function of the number of iterations in the ADMM algorithm (Additional file 1: Fig. S2) confirms that the total variation loss diminishes as the conductance steps become more distinguishable. Thus, the proposed method excels in reconstructing single-molecule measurement data, emphasizing clear changes in state. Subsequently, further validation was conducted at λ = 1, where the steps are distinctly reconstructed.

**Fig. 3:**
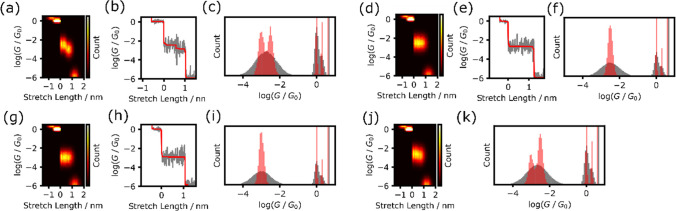
Histogram analysis of simulated data for Class 1 (**a–c**), Class 2 (**d–f**), Class 3 (**g**–**i**), and dataset constructed from the three classes with equal appearance rate (**j**, **k**). **a**, **d**, **g**, **j** 2D conductance-stretch length 2D histograms for Class 1 (**a**), Class 2 (**d**), Class 3 (**g**), and dataset constructed from the three classes (**j**). **b**, **e**, **h** Raw(gray) and reconstructed (red) conductance trace of Class 1 (**b**), Class 2 (**e**), Class 3 (**h**). **c**, **f**, **i**, **k** Conductance histograms constructed from raw (gray) and reconstructed (red) traces

The observed conductance often exhibits variability when using single-molecule measurements. Determining single-molecule conductance typically involves the construction of a conductance histogram using numerous conductance traces[3, 4]. In this study, histogram analyses were conducted for three distinct classes of traces, as shown in Fig. 3. The 2D conductance-stretch length histograms of the three classes before TVD processing are shown in Figs. 3a, d, and g. For Class 1, the raw signal displays two plateaus, yet the resulting histogram exhibits only a single broad peak. Conventional single-molecule analyses using typical histograms may fail to distinguish between the two states. Conversely, the histograms derived from the TVD-reconstructed traces distinctly reveal two peaks, underscoring the efficacy of this method as a preprocessing step in differentiating between multiple conduction states. Even when the conductance trace displays a single plateau, as observed in Classes 2 and 3 (Figs. 3d–i), the proposed TVD-based method generates distinct plateaus in the histograms constructed using the reconstructed traces. These refined histograms significantly contribute in accurately determining the conductance. In instances where the BJ method reveals two conduction states, the observations encompass traces displaying both plateaus or those exhibiting only one plateau. In our analysis, we assumed a dataset comprising traces from Classes 1, 2, and 3, each with an equal appearance rate. The distinguishability between the two states is clear in the 2D histogram (Fig. 3j) and resultant conductance histograms (Fig. 3k). The total variation-denoised histogram exhibits two distinct peaks, underscoring the applicability of the proposed method to datasets with various types of conductance traces.

**Fig. 4:**
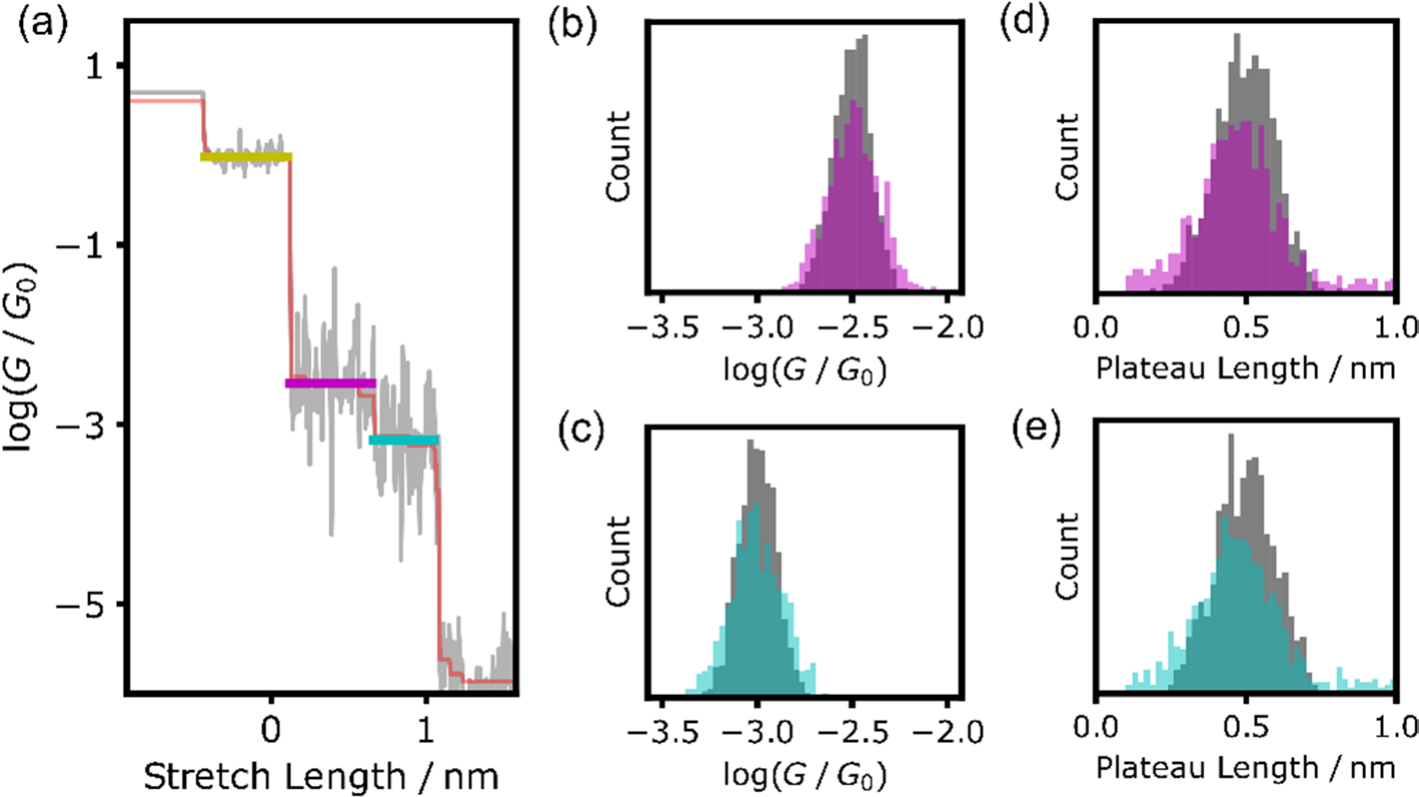
Result of Plateau detection analysis **a** Example of detected plateaus. Three plateaus (yellow, magenta, cyan) are detected based on TVD reconstructed trace (red). Gray line is raw signal. **b**, **c** Histograms of the plateau conductances of high- and low-conductance states for (**b**, **c)**, respectively. **d**, **e** Histograms of lengths of the detected plateaus of high- and low-conductance states for **d**, **e**, respectively. Gray histograms in (**b–e**) represents conductance and plateau length in ground truth conductance traces. Magenta and cyan histogram represents detected plateau of high- and low-conductance state, respectively

In contrast to conventional smoothing methods, the TVD-based approach demonstrated distinctive step-like conductance transitions. This method not only facilitates denoising and precise conductance determination but also plateau analysis, as shown in Fig. 4a. Although previous studies analyzed plateau lengths [33], our method offers the advantage of defining plateaus without explicitly specifying the conductance region. This enables the determination of the plateau length and conductance under consistent conditions, devoid of arbitrariness, and is particularly beneficial in delineating multiple conduction states with subtle differences. Further details regarding the definition of the plateau region may be found in the Additional file 1. Using TVD-based analysis, plateau analysis successfully detects high- and low-conductance states with a 92% detection rate, based on 1000 Class 1 traces. Even when the assigned plateaus are counted for the high- or low-conductance state during validation, this method demonstrates a high detection rate. Its effectiveness in identifying both conduction states, even in traces with fuzzy boundaries owing to noise, underscores its robustness in state identification. The histograms revealing the conductances and lengths of the detected plateaus (Fig. 4b–e) show respective average log(*G*/* G*_0_) values of – 2.49 and – 3.00 for high- and low-conductance states. The log conductance values of the high- and low-conductance states exhibit slight differences of 0.08 from the ground truth values. Average plateau lengths of 0.52 and 0.53 nm are observed. The histograms are consistent with the original conductance and plateau length. Estimating the plateau lengths is critical in inferring the stabilities of individual single-molecule junction states and molecular junction structures. Furthermore, identifying regions of identical conduction states via plateau analysis facilitates the evaluation of the noise magnitude within each conduction state [26, 35]. In a single-molecule junction, the conductance and its variation are crucial in representing the junction-specific state. The proposed method enables the evaluation of the conductance variation within each conduction state during BJ measurements, revealing novel insights into molecular junctions.

**Fig. 5:**
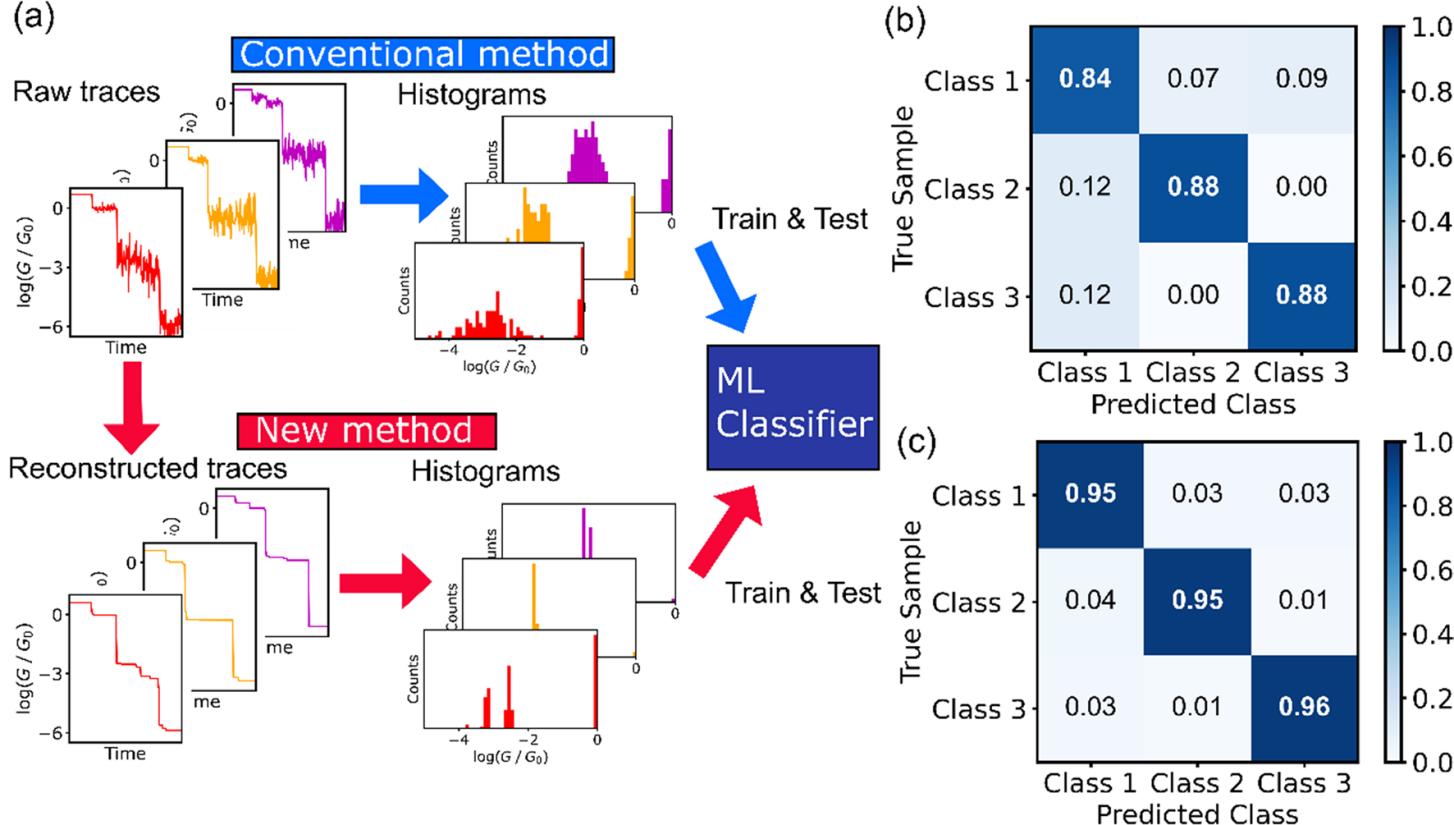
**a** Single-molecule supervised machine learning classification scheme of conventional and proposed method. **b**, **c** Confusion matrices of three classes classification using conventional method (**b**) and proposed method (**c**).

Recently, efforts to enhance the discrimination accuracy of measured single-molecule data emerged via the application of supervised machine learning techniques [10, 20, 24–27, 36]. Unlike focusing solely on a single average conductance, machine learning scrutinizes conductance variation, augmenting single-molecule discrimination. Our method serves as a viable preprocessing step for use in supervised machine learning applications. Figure 5a shows a schematic comparison between the conventional identification method for single-molecule traces and the proposed method. In the conventional approach, conductance histograms derived from individual traces are used as features in supervised machine learning classification [24, 27]. However, in this study, we first conducted denoising, using the reconstructed traces as inputs for classification in the same manner. Conductance traces belonging to Classes 1, 2, and 3 were discriminated using the conventional and TVD methods. The detail of supervise machine learning is described in Additional file 1. The confusion matrices displaying the classification results obtained using the conventional and proposed methods are shown in Fig. 5b and c, respectively. In the conventional method, the misclassification of Class 1, which is characterized by two plateaus, occurs more frequently, primarily owing to noise hindering the differentiation between the two conduction states. The classification performance was evaluated using one of the performance indicators, i.e., the *F*-measure, which is defined as the harmonic mean of sensitivity and specificity. The respective *F*-measure scores of the conventional and proposed methods are 0.87 and 0.95. Machine learning classifies the histograms based on individual traces as features, thus benefiting from the improved classification accuracy derived from the distinct histograms generated via TVD-based denoising. Our developed method not only enhances the classification accuracy but also serves as an effective preprocessing step for use in machine learning applications.

## Conclusion

In summary, the TVD method introduced in this study clarified the plateaus and transitions within the conduction states derived from the conductance traces. By revealing the conductances and plateau lengths while enhancing trace discrimination, this method contributed significantly in unraveling conductance transitions within molecular junctions with multiple conduction states. Its application should provide previously undisclosed insights into the dynamics of changes within single-molecule junctions.

### Supplementary Information

**Additional file 1**. Supplementary Information.

## Data Availability

The datasets generated and/or analyzed during the current study are available from the corresponding author on reasonable request.

## Code availability

The source codes are available from the corresponding author on reasonable request.
