# Supplementary material for: Total variation denoising-based method of identifying the states of single molecules in break junction data
Source: Discov Nano. 2024 Jan 29;19(1):20. doi: 10.1186/s11671-024-03963-4 (PMC10825082; doi:10.1186/s11671-024-03963-4)
Supplement: Supplementary file 1 — Additional file 1. Supplementary Information. [file 11671_2024_3963_MOESM1_ESM.pdf]

Supplementary Information for

Total variation denoising-based method of identifying the states of single molecules in break junction data

Yuki Komoto[1,2,3]*, Jiho Ryu[1], Masateru Taniguchi[1]

[1]SANKEN, Osaka University, 8-1 Mihogaoka, Ibaraki, Osaka, 567-0047, Japan
[2]Artificial Intelligence Research Center, Osaka University, Ibaraki, Osaka 567-0047, Japan
[3]Integrated Frontier Research for Medical Science Division, Institute for Open and Transdisciplinary Research Initiative (OTRI), Osaka University, Ibaraki, Osaka 567-0047, Japan

Correspondence to: komoto@sanken.osaka-u.ac.jp

Table of Contents

1. Dependence of regularization parameter λ................................ 2
2. Iteration dependence of reconstruction results 3
3. Details of analysis..4

1. Dependence of regularization parameter λ

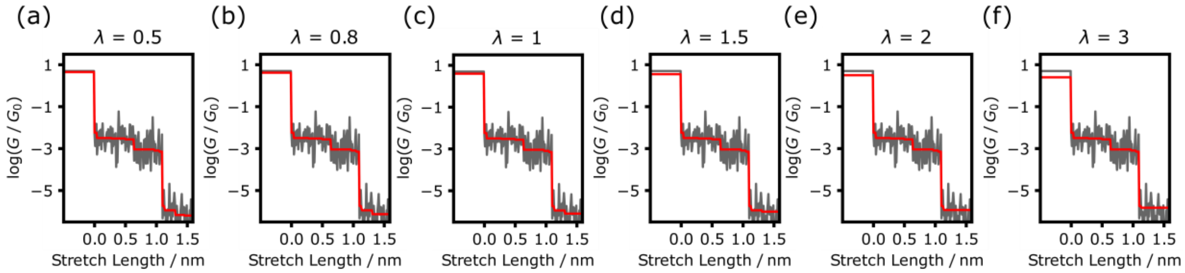

Figure S1. (a-f) Total variation denoised conductance traces with regularization parameter λ of 0.5, 0.8, 1, 1.5, 2, and 3, respectively. Reconstructed traces with different λ in range 0.5-3 exhibit no difference in molecular conductance region.

2. Iteration dependence of reconstruction results

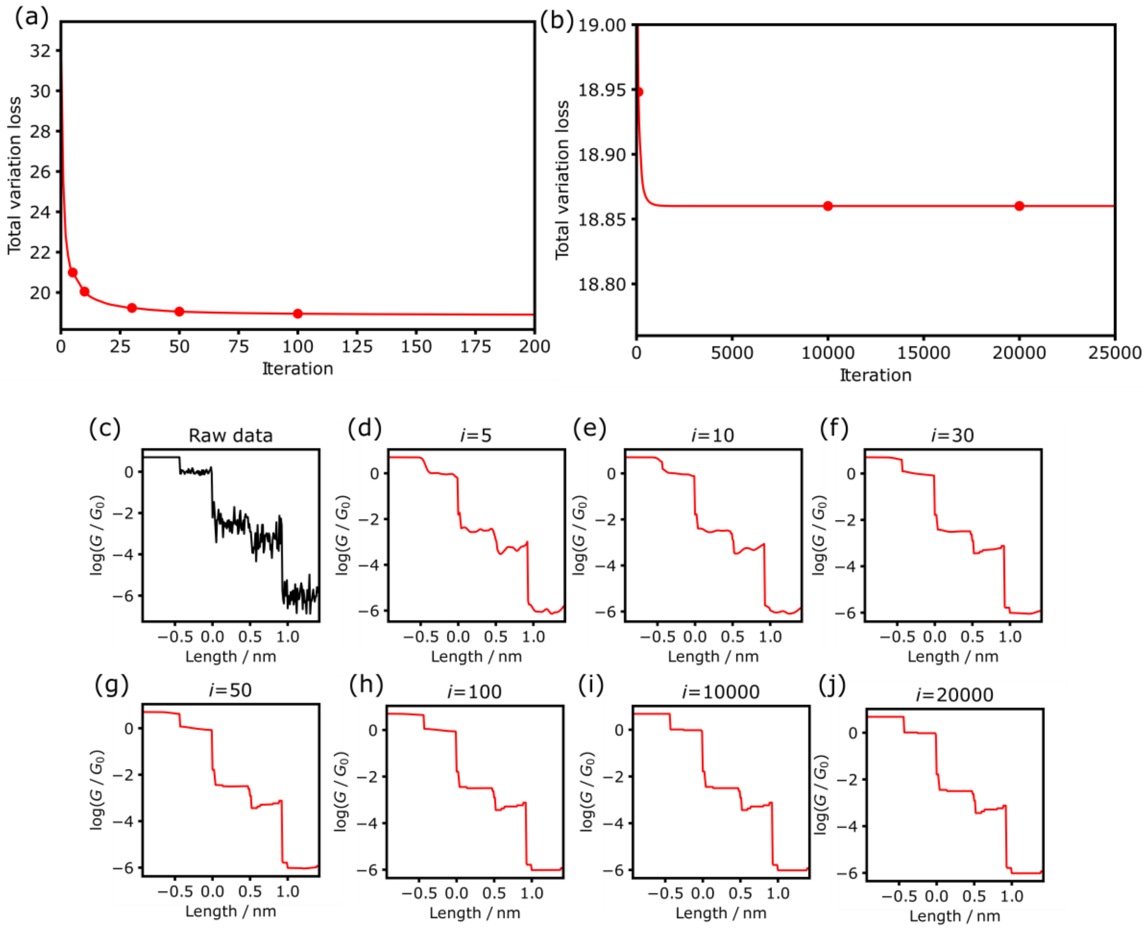

Figure S2. (a, b) Iteration dependence of total variation loss, Equation (1) in main text. The two figure a,b is different range of same analysis. Red dots denote the points which the reconstructed traces are shown in (d-j). (c) Analyzed raw conductance trace. (d-j) Reconstructed traces after iteration number 5,10,30,50,100,10000, and 20000, respectively.

In ADMM algorithm, the reconstruction is performed by iteration calculation. In this study, the max iteration number is 10000, where reconstruction trace change little.

3. Details of Analysis

plateau detection

The following analysis is performed on the reconstructed signals. First, plateaus were extracted as region which log conductance difference is smaller than the threshold of 0.1. The region with more than 10 data points length were adopted as plateaus. Then, two plateaus were merged if adjacent plateaus had a log conductance difference of less than 0.3 and a data interval of 2 points or less.

Details of supervised machine learning

The raw and reconstructed conductance trace were converted into histograms which log (G / G_0) range is from -5 to 0, 50 bins. The histograms were trained as feature into random forest classifier in sci-kit learn package. The hyper parameter of the classifier is default value. The classification results were evaluated with 10-fold cross validation. 900 traces for each class were trained and rest 100 traces were predicted. The average of 10-times classification is shown in confusion matrices in main text.
